# Bridging the gap in nutrition assessment: Barriers and knowledge impact of an educational session on body composition and energy metabolism

**DOI:** 10.1002/ncp.70092

**Published:** 2026-02-02

**Authors:** Camila E. Orsso, Anne Caretero, Homa Ghomashchi, Marlis Atkins, Thiago G. Barbosa‐Silva, Carlota Basualdo‐Hammond, Diana Cardenas, Stephanie Chevalier, M. Cristina Gonzalez, Leah Gramlich, Harriët Jager‐Wittenaar, Nicole Kiss, Sarah A. Purcell, Maira Quintanilha, Satnam Sidhu, Puneeta Tandon, Mei Tom, Carla M. Prado

**Affiliations:** ^1^ Department of Agricultural, Food, and Nutritional Science University of Alberta Edmonton Alberta Canada; ^2^ Nutrition Services Alberta Health Services Edmonton Alberta Canada; ^3^ Department of General Surgery, Faculty of Medicine Federal University of Pelotas Pelotas Brazil; ^4^ Nutrition Unit, Gustave Roussy Villejuif France; ^5^ School of Human Nutrition McGill University Sainte‐Anne‐de‐Bellevue Quebec Canada; ^6^ Research Institute of the McGill University Health Centre McGill University Montreal Quebec Canada; ^7^ Postgraduate Program in Nutrition and Food Federal University of Pelotas Pelotas Brazil; ^8^ Pennington Biomedical Research Center Louisiana State University System Baton Rouge Louisiana USA; ^9^ Department of Medicine, Division of Gastroenterology (Liver Unit) University of Alberta Edmonton Alberta Canada; ^10^ Department of Diabetes, Obesity, and Nutrition Strategic Clinical Network Alberta Health Services Calgary Alberta Canada; ^11^ Research Group Healthy Ageing, Allied Health Care and Nursing Hanze University of Applied Sciences Groningen Netherlands; ^12^ Department of Gastroenterology and Hepatology Dietetics Nijmegen Netherlands; ^13^ Department of Physiotherapy, Human Physiology and Anatomy, Faculty of Physical Education and Physiotherapy, Research Unit Experimental Anatomy Vrije Universiteit Brussel Brussels Belgium; ^14^ Institute for Physical Activity and Nutrition Deakin University Geelong Melbourne Australia; ^15^ Department of Medicine University of British Columbia Vancouver British Columbia Canada; ^16^ Centre for Chronic Disease Prevention and Management, Faculty of Medicine University of British Columbia Kelowna British Columbia Canada; ^17^ School of Health and Exercise Sciences University of British Columbia Okanagan Kelowna British Columbia Canada; ^18^ BC Cancer Victoria Victoria British Columbia Canada

**Keywords:** body composition, diet, energy metabolism, healthcare, implementation, methods, nutrition assessment, nutrition care process, nutritionists, practice change

## Abstract

**Background:**

Body composition and energy metabolism assessments are central to nutrition care but remain underused. This study evaluated dietitians’ access, use, education, and beliefs toward these assessments, and the impact of an educational session.

**Methods:**

Dietitians (≥1 year experience) within Alberta Health Services completed online surveys on their access, use, education, confidence, and clinical protocol awareness regarding body composition and energy metabolism assessments. Participants attended a 4‐h virtual educational session on these topics, with presession and postsession knowledge recall compared using Wilcoxon signed rank test.

**Results:**

We surveyed 36 participants (97% women), primarily from inpatient care (36%) or oncology (31%). Most (86%) never used body composition methods, despite access to computerized tomography (CT; 31%), ultrasound (US; 25%), bioelectrical impedance analysis (BIA; 6%), and dual‐energy x‐ray absorptiometry (DXA; 3%). Although 67% received body composition education (47% BIA, 31% DXA, 22% CT, 8% US), 64% lacked confidence. Protocol awareness was 67% for malnutrition and 8% for sarcopenia. Most never (58%) or occasionally (19%) used energy metabolism methods; Q‐NRG (COSMED; 28%) and standard metabolic carts (19%) were the most available devices. Although 67% received energy metabolism education (53% metabolic cart, 31% Q‐NRG), 50% were not confident in using these methods. After the educational session, body composition knowledge recall improved (median, +20% [IQR, 10–30]; *P* < 0.001), whereas energy metabolism scores were unchanged (*P* = 0.530).

**Conclusion:**

Despite prior education, dietitians used body composition and energy metabolism assessments inconsistently because of limited access, insufficient applied knowledge, lack of protocols, and low confidence. These results underscore the need to strengthen curriculum and professional development to support adoption.

## Graphical abstract

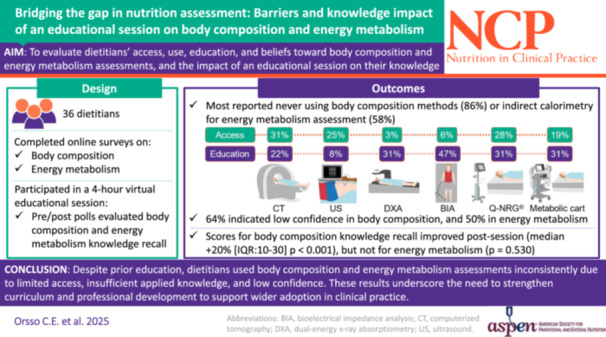

## INTRODUCTION

Alterations in body composition and energy metabolism are common in individuals with acute and chronic diseases, as well as in aging populations.^1^, ^2^, ^3^, ^4^ As such, the use of methods to assess body composition and energy metabolism is becoming increasingly important for evaluating nutrition status across the continuum of care.

Although much of the clinical focus has centered on low muscle mass, an important phenotypic criterion for malnutrition^5^, ^6^ and a predictor of adverse health outcomes,^1^, ^7^ unfavorable changes in fat mass also have implications for health and disease.^8^, ^9^ In parallel, altered energy metabolism is prevalent in many clinical populations,^10^, ^11^ making accurate assessment of caloric requirements essential to inform nutrition intervention. Indirect calorimetry (IC), widely used to estimate components of energy metabolism particularly in intensive care units, captures individual variability in energy needs and helps prevent underfeeding or overfeeding.^12^, ^13^ Given their clinical utility, determining when and how to integrate body composition and energy metabolism assessments into the nutrition care process (NCP) could enhance tailored nutrition care.^14^ These tools can help identify nutrition‐related conditions, predict clinical outcomes, and support the development of targeted treatment strategies.^12^, ^15^, ^16^, ^17^, ^18^ Despite their value, these assessments remain underused, underscoring the urgent need for a systematic approach to implement them in routine practice.^19^, ^20^, ^21^

Prior research has identified several barriers to integrating body composition and energy metabolism assessments into the NCP, including limited access to equipment, insufficient practitioner expertise, and absence of standardized procedures.^13^, ^20^, ^21^, ^22^, ^23^ Low rates of malnutrition coding using *International Classification of Diseases* codes^24^ suggest inconsistent application of body composition assessments, particularly within workflows that incorporate muscle evaluation as recommended by frameworks such as the Global Leadership Initiative on Malnutrition (GLIM).^5^, ^6^ Furthermore, most existing studies have focused on specific body composition methods or select clinical settings, with limited research conducted on the implementation of IC for energy metabolism assessment. Understanding and addressing the barriers healthcare professionals face in routine clinical practice is essential for successfully integrating these assessments into care pathways.^25^ As primary providers in the management of nutrition‐related conditions through the application of the NCP, dietitians play a central role in this integration. Notably, although focused on dietitians, these barriers are also relevant to other healthcare professionals involved in assessment and intervention of low muscle mass and related conditions (eg, malnutrition, sarcopenia, sarcopenic obesity, cachexia) across inpatient and outpatient settings. Accordingly, this study aimed to evaluate dietitians’ access to, use of, education on, and beliefs about body composition and energy metabolism assessments. We also examined their baseline knowledge and evaluated the effectiveness of a virtual educational session as a training module.

## METHODS

### Study design and population

This study presents findings from phase 1 of a two‐phase implementation planning project, aimed at identifying a practical pathway for integrating body composition and energy metabolism assessment methods across six selected clinical practice areas.^26^ Phase 1 involved a synchronous, 4‐h virtual educational session held in January 2023, featuring six lectures delivered by practitioners, including dietitians and medical doctors, and by academics and/or research professionals with specialized expertise in nutrition assessment within daily practice. Details of the session are available in the study protocol.^26^ In addition to attending the educational session, participants provided data on demographics, professional experience, and baseline capacities in these assessments. They also completed pre–educational session and post–educational session evaluations.

Participants were dietitians employed by Alberta Health Services (AHS) with at least 1 year of experience working in oncology, liver diseases, kidney diseases, critical care, weight management/bariatrics, or inpatient care (medicine and surgery). Additional eligibility criteria included availability to attend a 4‐h educational session and a 4‐h virtual group meeting during working hours on preestablished dates. Potential participants were identified by AHS Nutrition Services leads from each selected practice area and geographical zone. Recruitment emails were sent, and those who completed a sign‐up form via Research Electronic Data Capture (REDCap) were contacted by the researcher team with further study details.

This study was approved by the University of Alberta Health Research Ethics Board (Pro00123353), and operational and administrative approvals were received from AHS and Northern Alberta Clinical Trials and Research Centre (NACTRC). Written informed consent was obtained from all participants.

### Baseline surveys

Before the educational session, participants completed two researcher‐designed surveys administered on REDCap, available in our previous publication.^26^ One of these surveys included 17 items and collected information on participants’ professional experience as dietitians (both current and past practice areas), as well as demographics such as age, gender identity, ethnic origin (race), and education level. Participants’ AHS geographical zones were also recorded to ensure representation across diverse practice settings within Alberta. Reporting by zone is relevant given potential differences in clinical resources, access to assessment tools, and regional implementation support, which may influence the integration of such assessments into practice. The second survey, a 24‐item baseline competency assessment, evaluated participants’ access to and use of body composition and energy metabolism methods (particularly IC, including standard metabolic carts, the new‐generation Q‐NRG [COSMED] metabolic monitors, and others) in their routine practice, as well as prior education/training and beliefs regarding these assessments.^26^ Although components of energy metabolism can be evaluated using multiple methods, IC is considered the gold standard in clinical settings because of its accuracy, noninvasive nature, and feasibility for bedside use in both spontaneously breathing and mechanically ventilated patients.^12^ The frequency of using these methods was measured using a 5‐point scale: never, rarely, sometimes, very often, and always. For simplicity, “rarely” is referred as infrequent use, “sometimes” as occasional use, and both “very often” and “always” as frequent use. Additional questions explored participants’ awareness of existing workplace protocols for screening and/or diagnosing malnutrition, sarcopenia, and sarcopenic obesity. The baseline competency survey was pilot tested by four dietitians affiliated with our research laboratory.^26^

### Pre–educational session and post–educational session evaluation

To assess participants’ knowledge recall, we developed a 10‐item multiple‐choice quiz focused on body composition, structured into five thematic sets.^26^ According to the Bloom's original taxonomy, “knowledge” represents the lowest level of cognitive processing and corresponds to “remembering” in the revised taxonomy,^27^ which accurately reflects the intended focus of our evaluation. Each set included two questions about body composition fundamentals and specific assessment methods: computerized tomography (CT), dual‐energy x‐ray absorptiometry (DXA), bioelectrical impedance analysis (BIA), and ultrasound (US). One additional set of two multiple‐choice questions assessed knowledge of energy metabolism fundamentals. Question sets were administered immediately before and after each lecture using the Zoom (Zoom Communications) polling feature, with 45–60 s allocated per question. Polls were monitored in real time to ensure completion and were closed once all participants had submitted their responses. After the educational session, a polling report was generated from Zoom. Because the meeting settings were configured to be nonanonymous, as approved by our ethics board, we were able to record each participant's individual responses to the questions. Each correct answer received 1 point; incorrect or unanswered questions scored 0. Thus, the maximum possible score (ie, total knowledge recall score) for body composition was 10 and for energy metabolism was 2. Scores were analyzed as cumulative totals, and percentage improvement was calculated using the formula: (posttest score – pretest score) ÷ (highest possible knowledge recall score × 100). The proportion of participants with improved scores for each set of questions on body composition fundamentals and methods was computed.

### Statistical analysis

Descriptive statistics were used to summarize participants’ demographics, professional experience, and baseline competencies. Continuous variables were reported as medians and interquartile ranges (25th–75th percentiles), and categorical variables were reported as percentages. The distribution of pretest and posttest knowledge recall scores was assessed using the Shapiro‐Wilk test. Because the data were not normally distributed, nonparametric tests were applied. Changes in body composition and energy metabolism knowledge recall scores from pretest to posttest were analyzed using Wilcoxon's signed rank test. The proportion of participants achieving a total body composition knowledge recall score of 7 or higher was compared between pretests and posttests using Fisher exact test. A threshold of 7 was pragmatically selected, as it was considered to represent a score high enough to reflect adequate knowledge, distinguishing clear understanding from limited or basic recall. Binomial tests were performed to determine whether the proportion of participants who improved their knowledge recall scores on body composition fundamentals and selected methods differed significantly from the 0.5 probability expected by chance. Sensitivity analyses evaluated the impact of prior education/training and confidence levels on the percentage improvement of the total knowledge recall scores using the Mann‐Whitney *U* test for group comparisons. Statistical analyses were conducted in JASP (v.0.17).

## RESULTS

### Participants’ demographics and professional experience

Fifty‐five dietitians were invited to participate. Of these, 40 accessed the sign‐up form, and 36 completed the online surveys (65.5% response rate) and were included in the study (Table 1). No notable differences in age or gender were observed between those who accessed the form and those who were included in the analysis. Participants had a median age of 40.0 years (IQR, 37.0–45.3), 97.2% were women, 88.9% identified as Caucasian/White, and 77.8% held a bachelor's degree as their highest level of education. Most (75%) had >10 years of clinical experience as dietitians. Although all six selected practice areas were represented, the largest proportions worked in inpatient care (36.1%) and oncology (30.6%). Notably, 41.7% reported working in two or more practice areas. The duration of experience in each practice area is shown in Figure S1. Most participants were from the AHS Edmonton Zone (50.0%), followed by the Calgary Zone (22.2%) within Alberta, Canada.

**Table 1:** Participants’ characteristics.

Characteristics	Total (*N* = 36)
Age, median (IQR), years	40.0 (37.0–45.3)
Gender identity	
Women	35 (97.2)
Men	1 (2.8)
Ethnic origin/race^a^	
Caucasian/White	32 (88.9)
Black	2 (5.6)
Chinese	2 (5.6)
Latin American	1 (2.8)
Arab	1 (2.8)
Educational level	
Bachelor's degree	28 (77.8)
Master's degree	6 (16.7)
Academic doctorate degree	1 (2.8)
Professional doctorate degree	1 (2.8)
Years of experience as a dietitian	
0–5	2 (5.6)
6–10	7 (19.4)
11–15	6 (16.7)
16–20	9 (25.0)
20+	12 (33.3)
Current practice area^a^	
Oncology	11 (30.6)
Liver disease	5 (13.9)
Kidney disease	8 (22.2)
Critical care	9 (25.0)
Weight management/bariatric	5 (13.9)
Inpatient care	13 (36.1)
Other	8 (22.2)
AHS geographical zones	
Edmonton	18 (50.0)
Calgary	8 (22.2)
Central	4 (11.1)
South	3 (8.3)
North	2 (5.6)
Provincial	1 (2.8)

*Note*: Values are presented as *n* (%) unless otherwise specified.

Abbreviation: AHS, Alberta Health Services.

^a^ Participants were able to select more than one option for the question; therefore, totals exceed 100%.

### Baseline competency in body composition

**Figure 1:**
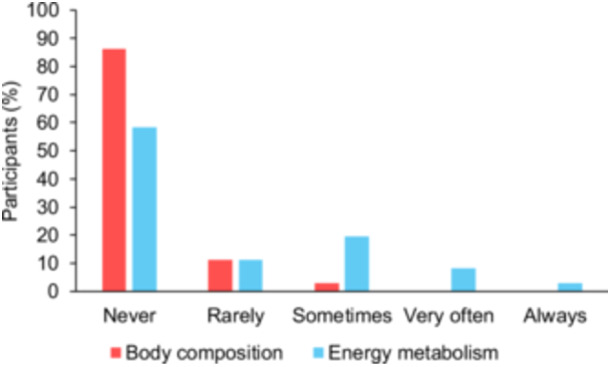
Participants’ use of body composition and energy metabolism assessments in routine clinical practice (*N* = 36).

Most participants (86.1%) reported never using body composition methods in their current practice (Figure 1), despite the reported access to CT (30.6%), US (25.0%), BIA (5.6%), and DXA (2.8%) in their workplaces. Additionally, 80.6% did not use anthropometric methods (ie, body circumferences and skinfolds). Among those who occasionally (5.6%) or infrequently (11.1%) used anthropometry, the most measured parameters included waist and calf circumferences.

**Figure 2:**
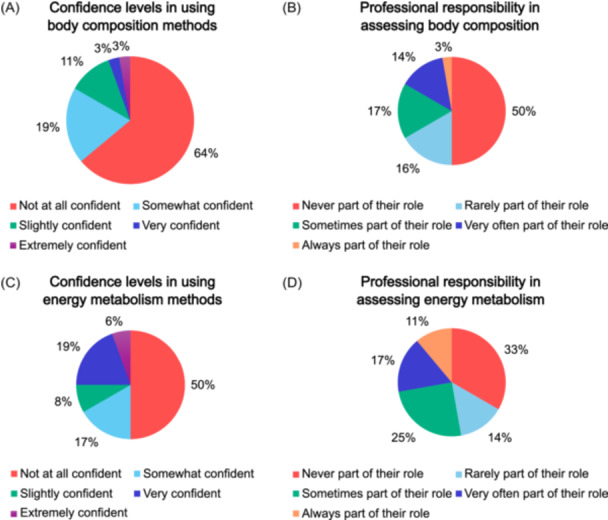
Participants’ confidence and perceived professional responsibility in assessing body composition and energy metabolism (*N* = 36). (A) Confidence levels in using body composition methods. (B) Perceived professional responsibility for assessing body composition in routine practice (indication of how often body composition assessment was part of their professional role). (C) Confidence levels in using energy metabolism methods. (D) Perceived professional responsibility for assessing energy metabolism in routine practice (indication of how often energy metabolism assessment was part of their professional role).

Nearly 67.0% of participants had received education in body composition assessment, primarily in BIA (47.2%), DXA (30.6%), CT (22.2%), and US (8.3%). Sources of education included as part of university coursework (44.4%), conferences (19.4%), in‐person workshops (13.9%), and online courses (8.3%). An additional 13.9% reported gaining knowledge through other means, such as research studies, certifications, or peer education. However, 63.9% reported lacking confidence in using such methods, and for 50.0%, body composition assessment had never been part of their professional role (Figure 2). Most participants (66.7%) were aware of a screening and/or diagnostic protocol for malnutrition in their workplace; however, awareness was much lower for protocols related to sarcopenia (83.3% reported no such protocol and 8.3% were unsure) and sarcopenic obesity (91.7% reported no protocol and 8.3% were unsure). Awareness by practice area is presented in Figure S2.

### Baseline competency in energy metabolism

Over 58.0% of participants reported never using IC for energy metabolism assessment in their current practice, whereas 19.4% indicated occasional use (Figure 1). The most commonly available methods in participants’ workplace were Q‐NRG devices (27.8%) and standard metabolic carts (19.4%). Infrequent or occasional use of portable IC devices (eg, MedGem [Microlife Medical Home Solutions]) was reported by 8.3% and 2.8% of participants, respectively.

Most participants (66.7%) had received education on energy metabolism, with specific training reported for standard metabolic cart (52.8%), Q‐NRG (30.6%), and portable IC devices (8.3%). Education sources included as part of university coursework (33.3%), conferences (27.8%), in‐person workshops (27.8%), and online courses (22.2%). An additional 27.8% reported gaining knowledge through other means such as self‐direct studies, certifications, peer education, or workplace training.

Confidence in using energy metabolism assessment methods varied: 50.0% reported not feeling confident, 16.7% felt somewhat confident, and 19.4% expressed high confidence. For 33.3% of participants, assessing energy metabolism had never been part of their professional role (Figure 2).

### Pre–educational session and post–educational session evaluation

**Figure 3:**
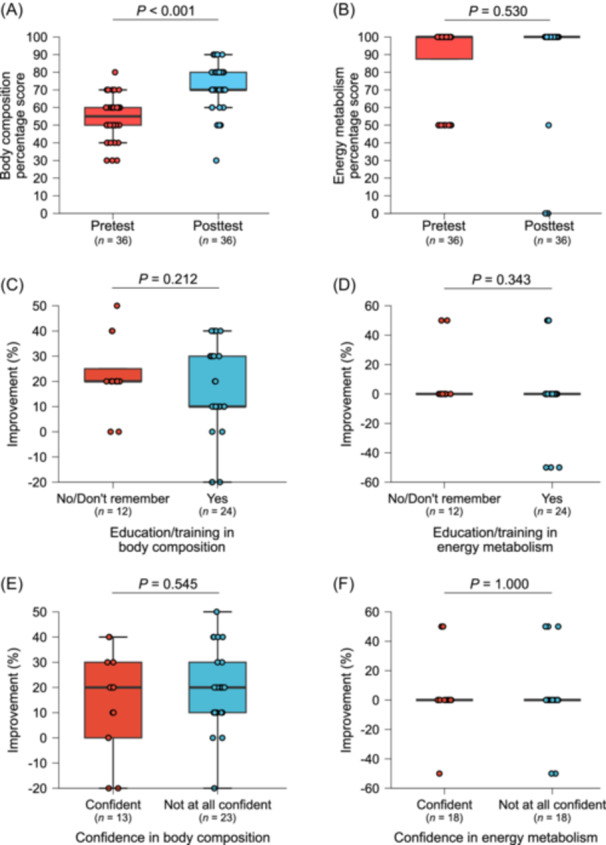
Impact of a virtual education session on participants’ knowledge recall of body composition and energy metabolism (*N* = 36). (A) Pretest and posttest percentage scores for total body composition knowledge recall. (B) Pretest and posttest percentage scores for total energy metabolism knowledge recall. (C) Percentage improvement in knowledge recall scores for body composition stratified by prior education and/or training. (D) Percentage improvement in knowledge recall scores for energy metabolism stratified by prior education and/or training. (E) Percentage improvement in knowledge recall scores for body composition stratified by self‐reported confidence. (F) Percentage improvement in knowledge recall scores for energy metabolism stratified by self‐reported confidence.

All participants completed the knowledge recall quiz. The total knowledge recall score on body composition significantly improved, rising from a median of 5.5 (5.0–6.0) points on the pretest to 7.0 (7.0–8.0) points on the posttest, representing a median increase of 20.0% (10%–30%) (*P* < 0.001). Initially, 19.4% scored ≥7.0 on the pretest, increasing to 80.6% on the posttest (*P* = 0.116) (Figure 3). Participants’ understanding of CT, DXA, BIA, and US improved after the educational session; however, the proportion of participants who showed improvement did not differ significantly from those who did not for these body composition methods (Table 2). In contrast, scores on the two questions assessing body composition fundamentals remained unchanged, likely reflecting high baseline knowledge, as 88.9% and 94.4% of participants had already answered these questions correctly before the session, with only 11.1% showing improvement (*P* < 0.001).

**Table 2:** Pretest and posttest knowledge recall scores on BC fundamentals and methods, as well as proportion of participants with improved scores.

	Knowledge recall scores			Participants with improved scores	
**Topics**	**Pretest**	**Posttest**	***P*** **value** a	***n*** **(%)**	***P*** **value** b
BC fundamentals	2.0 (2.0–2.0)	2.0 (2.0–2.0)	0.072	4 (11.1)	<0.001
CT	0.0 (0.0–1.0)	1.0 (0.0–1.0)	0.008	12 (33.3)	0.065
DXA	1.0 (0.0–1.0)	1.0 (1.0–2.0)	0.007	20 (55.6)	0.618
BIA	1.0 (1.0–2.0)	2.0 (1.0–2.0)	0.042	14 (38.9)	0.243
US	1.0 (1.0–2.0)	2.0 (2.0–2.0)	<0.001	19 (52.8)	0.868

*Note*: Pretest and posttest values are reported as median (IQR).

Abbreviations: BC, body composition; BIA, bioelectrical impedance analysis; CT, computed tomography; DXA, dual‐energy x‐ray absorptiometry; US, ultrasound.

^a^ Wilcoxon signed rank test.

^b^ Binomial test, proportion tested against value of 0.5.

Similar findings were observed for energy metabolism total knowledge recall scores, which did not change significantly from pretest to posttest (*P* = 0.530), as most participants achieved the maximum score at baseline (Figure 3). There were no differences in percentage change of total knowledge recall scores for either body composition or energy metabolism assessments across participants grouped by prior education and confidence levels (Figure 3).

## DISCUSSION

Body composition and energy metabolism assessments are essential for evaluating nutrition status, assessing the effectiveness of nutrition interventions, and predicting disease risk and clinical outcomes.^12^, ^15^, ^16^, ^17^, ^18^ Despite some dietitians having access to these methods and prior exposure through education, our findings indicate that they are not routinely conducted in clinical practice. We also found that participants’ knowledge of body composition significantly improved after the educational session, underscoring the effectiveness of structured training modules in enhancing knowledge recall. Incorporating these assessments into the NCP and ongoing education are therefore essential for advancing patient‐centered care.

Our results align with previous research, reporting limited integration of body composition and energy metabolism assessments in routine practice.^20^, ^21^, ^23^, ^28^ A primary barrier identified for current use and future implementation is the limited equipment access in the workplace. Consistent with prior studies,^20^, ^21^, ^28^ fewer than one‐third of participants reported access to at least one body composition method (CT, DXA, BIA, and US) or IC device (ie, standard metabolic cart and Q‐NRG). These specific methods were selected based on their accuracy, feasibility, potential availability, and alignment with the GLIM body composition guidelines for assessing the muscle mass phenotype in malnutrition diagnosis.^5^, ^6^ Nevertheless, barriers such as equipment costs, portability, patient compatibility, and training requirements^12^, ^15^, ^29^ may limit their accessibility and uptake in clinical settings. Future research should explore whether limited awareness of available workplace resources or uncertainty about how to access equipment contributes to the underuse of readily accessible clinical methods, such as US.

Another important barrier was the absence of assessment body composition protocols for nutrition‐related conditions. Most participants reported being unaware of existing protocols for screening and/or diagnosing sarcopenia and sarcopenic obesity, and nearly 40% were also unaware of protocols for malnutrition, particularly those working in weight management/bariatric and kidney disease clinics. This likely reflects limited implementation or dissemination of standardized procedures rather than the complete absence of nutrition assessment. Furthermore, findings are consistent with previous research involving medical professionals, where most lacked knowledge of sarcopenia diagnosis and relied on clinical judgment rather than on validated criteria, regardless of whether formal protocols were implemented.^20^ This gap is not necessarily a local limitation but may reflect the broader, ongoing effort to establish and disseminate widely accepted definitions and diagnostic protocols for these conditions across various clinical settings.^24^ Although surrogate anthropometric measures, such as calf circumference and mid‐arm muscle circumference, can be used when body composition methods are unavailable,^5^, ^6^ only a few participants reported occasional use of these measures. This suggests that even assessments requiring minimal tools remain underutilized in the absence of formal protocols. These results highlight the development and implementation of assessment protocols as a critical step toward integrating body composition assessments in routine practice. Such protocols provide a structured framework for healthcare professionals, promoting consistency and reliability of assessments within existing clinical workflows.

The inclusion of participants from multiple geographic regions enhances the generalizability of our findings across diverse clinical settings in our province. However, greater representation from urban centers (ie, AHS Edmonton and Calgary Zones) may reflect differences in resource availability and training opportunities that are not equally accessible in other areas, particularly in rural or underresourced settings. For instance, >8% of participants reported US training for body composition assessment, but this finding may have limited generalizability, as previous studies indicate that only 1%–6% of dietitians routinely use this method in practice.^30^, ^31^ These variations underscore the importance of context‐specific implementation strategies, especially in regions with limited access to body composition and energy metabolism assessment tools. Several barriers to delivering quality nutrition care have been identified in resource‐limited settings, including the absence of nutrition assessment procedures and nutrition care plans.^32^ Failure to routinely implement these assessments may limit the ability to identify patients at risk, personalize interventions, and improve clinical outcomes, ultimately exacerbating healthcare disparities and reinforcing the urgency of addressing this practice gap.

Dietitians’ beliefs toward body composition and energy metabolism assessments can substantially influence their integration into routine practice. A previous study identified common barriers for the use of body composition, including low confidence among dietitians and the perception that such assessments should be conducted by other professionals.^21^ Our findings reflect similar trends, although confidence levels related to energy metabolism assessments were slightly higher than those for body composition. While we did not directly explore the underlying motivations behind these beliefs and practices, nearly half of the participants reported no prior education or training in these assessments, an important factor that may have contributed to their perceptions. Cultural influences may also have played a role; for example, in Canada, sensitivity around discussions of body weight and size may discourage routine measurement practices. This is supported by qualitative findings reporting that Canadian dietitians were hesitant to use the term *malnutrition* with patients because of perceived stigma, and that patients often found the word unrelatable or extreme.^33^ These findings highlight a broader discomfort in communicating weight‐ or nutrition‐related diagnoses, suggesting that cultural attitudes may act as a barrier not only to terminology but also to routine physical assessment practices such as body composition and energy metabolism measurements. Additionally, <20% of participants scored ≥70% on the pretest body composition competency evaluation, suggesting limited understanding, particularly of such methods. These results emphasize the urgent need for capacity building among dietitians and other healthcare professionals in the use of these assessments, and for the integration of these components into future implementation strategies.

In our study, most participants reported obtaining education in body composition and energy metabolism assessments through university studies. However, despite this university‐level education, many did not incorporate these methods into routine practice and expressed low confidence in their use. This finding reflects that education is often theoretical and underscores the need to strengthen the curriculum of dietetics programs by enhancing content and providing more practical training opportunities. Interestingly, participants also reported gaining knowledge through peer education, self‐directed learning, and formal workplace training. Education remains a key driver for building confidence and awareness, as well as supporting the adoption of novel approaches within health systems.^34^, ^35^ Our virtual educational session effectively improved participants’ knowledge recall of body composition fundamentals. Although case studies were included to enhance the interpretation of body composition and energy metabolism findings, most lectures primarily focused on the comprehension level of Bloom's cognitive domain, without advancing to higher‐order skills such as application and analysis,^27^ owing to time limitation. To support the translation of this knowledge into clinical practice and foster leaners’ confidence, it is important to go beyond case‐based discussions and incorporate hands‐on, practical training that mirrors real‐world settings.^36^ A 3‐day practical training program has previously demonstrated improved knowledge and skill development in CT‐based body composition assessment among dietitians working with patients undergoing surgery, cancer treatment, or critical care.^37^ Furthermore, some participants reported the use of portable IC devices—these tools are known to have limited accuracy,^38^, ^39^, ^40^ and tools like Q‐NRG still require robust validation in diverse clinical settings. Although we have not further explored this finding, it underscores the opportunity to enhance awareness through targeted education on the strengths and limitations of available equipment, supporting informed decision‐making and appropriate use in clinical practice. Although not a finding of our study, decisions to purchase assessment tools may at times be influenced by marketing claims rather than evidence‐based information, underscoring the need for informed, critical evaluation of equipment capabilities.

A key strength of this project is the unique organizational structure of AHS, which includes a dedicated Nutrition and Food Services Department recognized nationally for implementing best practices in malnutrition screening, assessment, and standardized nutrition care. This department, led by leaders strongly committed to research, innovation, and advocacy, has been instrumental in supporting advancements in body composition and energy metabolism assessments. However, this strong infrastructure and centralized support may not be present in all healthcare systems, particularly in resource‐limited settings,^32^ which could affect the direct transferability of our implementation strategy. To promote broader applicability in upcoming study phases, we have proactively engaged representatives from different Canadian provinces, as well as international experts representing diverse healthcare systems and high‐income and mid‐income countries, which strengthens the potential for cross‐jurisdictional relevance and implementation.^26^ Notably, although this study focused on dietitians, broader implementation efforts should foster interdisciplinary collaboration to reinforce these assessments as shared responsibilities across care teams.

The study has limitations that should be discussed. The education session was delivered virtually, which may have posed challenges depending on participants’ familiarity with the Zoom platform. However, before the session, participants were asked about their comfort using Zoom features such as chat and polling, and 80% reported feeling comfortable or very comfortable (data not shown). Despite this, virtual settings can make it harder to sustain engagement and interpret audience cues, such as body language, which may have limited interaction between presenters and attendees.^41^ For knowledge recall evaluation, we used Zoom's polling feature with a limited response time (ie, 45–60 s) to minimize burden and provide immediate feedback. Although practical, the quiz format and time constraint may have limited participants’ ability to thoughtfully consider their responses, particularly during the pretest, possibly resulting in guesswork. Postsession evaluations were conducted immediately after each lecture, limiting the ability to assess longer‐term impacts on participants’ knowledge. Additionally, the knowledge recall quiz had not been previously validated and included only two questions related to energy metabolism fundamentals; these questions did not assess participants’ understanding of specific methods, thereby limiting the depth of our findings in this area. Furthermore, this study may not have captured all potential barriers to the current and future use of these assessments, and barriers were inferred from survey results; for example, time constraints or limited resources required to conduct these assessments in clinical settings. These additional factors will be explored in the next phase of our study through focus group discussions and SWOT (strengths, weaknesses, opportunities, and threats) analysis to develop a more comprehensive understanding of the challenges and enablers of implementing body composition and energy metabolism assessments within healthcare systems.

## CONCLUSION

Our study demonstrated that although most participating dietitians had received prior education in body composition and energy metabolism, these assessments were not consistently performed in routine clinical practice. Several barriers likely contributed to this limited integration, including restricted access to equipment, insufficient technical knowledge, absence of screening and diagnostic protocols for nutrition‐related conditions, and low confidence in conducting these assessments. Our virtual educational session effectively improved participants’ knowledge recall of body composition; however, further research is needed to determine whether practical, hands‐on training can also strengthen their skills and confidence for clinical application. Although our findings are context‐specific and may not be generalizable to other practice areas, centers, or healthcare systems, they provide valuable insight into key barriers, resources, and capacity building needed to support the implementation of assessments in the routine practice of dietitians and other healthcare professionals working across inpatient and outpatient settings. Together with the upcoming second phase of this project, these results will inform the development of a practical implementation pathway. Future research could explore the reasons behind participants’ perceptions that body composition and energy metabolism assessments are not a routine part of their professional role, and evaluate whether targeted educational interventions can help shift these perceptions.

## AUTHOR CONTRIBUTIONS

Camila E. Orsso and Anne Caretero contributed to the methodology, project administration/coordination, and interpretation of the data and drafted the original manuscript. Marlis Atkins, Carlota Basualdo‐Hammond, M. Cristina Gonzalez, Leah Gramlich, Puneeta Tandon, and Mei Tom contributed to the conceptualization and interpretation of the data and critically revised the manuscript. Homa Ghomashchi, Thiago G. Barbosa‐Silva, Diana Cardenas, Stephanie Chevalier, Harriët Jager‐Wittenaar, Nicole Kiss, Sarah A. Purcell, and Satnam Sidhu contributed to the interpretation of the data and critically revised the manuscript. Maira Quintanilha contributed to the methodology and interpretation of the data and critically revised the manuscript. Carla M. Prado contributed to the conceptualization, funding acquisition, supervision, methodology, and interpretation of the data, and critically revised the manuscript. All authors read and approved the final manuscript.

## CONFLICT OF INTEREST STATEMENT

Camila E. Orsso has received honoraria and speaker fees from Abbott Nutrition. Anne Caretero has received travel funding from Fresenius Kabi. Diana Cardenas has received honoraria and/or paid consultancy from Nutricia, Nestlé Health Science, and Fresenius Kabi. Diana Cardenas also serves as the ethics editor for *Clinical Nutrition*. M. Cristina Gonzalez has received paid consultancy from Nestlé Health Science Brazil and speaker fees from Abbott Nutrition and Nutricia. Leah Gramlich has received paid consultancy and speaker fees from Baxter, Fresenius Kabi, and Abbott. Nicole Kiss has received paid honoraria from Abbott Australasia and Nutricia. Maira Quintanilha is the owner of Quali Q Inc. Carla M. Prado has received honoraria and/or paid consultancy from Abbott Nutrition, Nutricia, Nestlé Health Science, and Novo Nordisk. The remaining authors declare no conflicts of interest.

## Supporting information

11.03.25_Supplementary_material.pdf.
