# Supplementary material for: Bridging the gap in nutrition assessment: Barriers and knowledge impact of an educational session on body composition and energy metabolism
Source: Nutr Clin Pract. 2026 Feb 2;41(3):859–70. doi: 10.1002/ncp.70092 (PMC13193499; doi:10.1002/ncp.70092)
Supplement: Supplementary file 1 — 11.03.25_Supplementary_material.pdf. [file NCP-41-859-s001.pdf]

Bridging the gap in nutrition assessment: barriers and knowledge impact of an educational session on body composition and energy metabolism

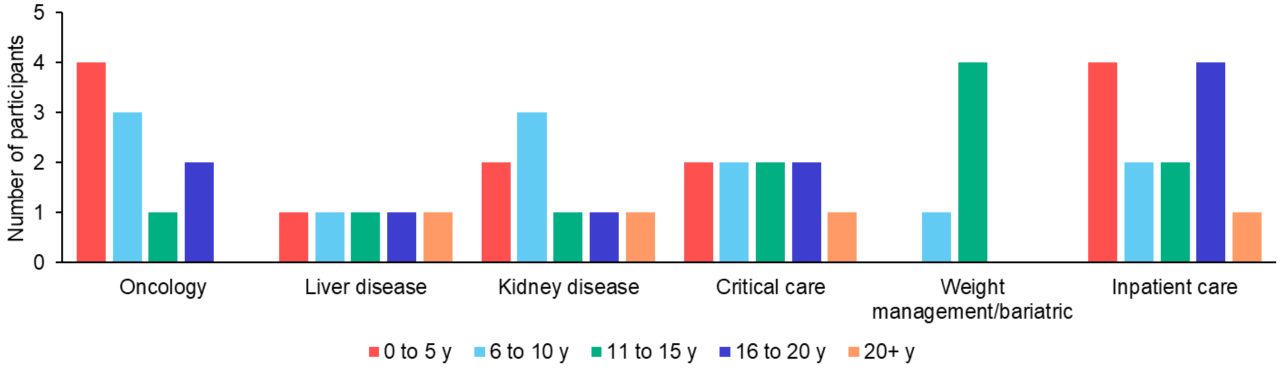

Figure S1. Participants' length of experience in selected practice areas.

Bridging the gap in nutrition assessment: barriers and knowledge impact of an educational session on body composition and energy metabolism

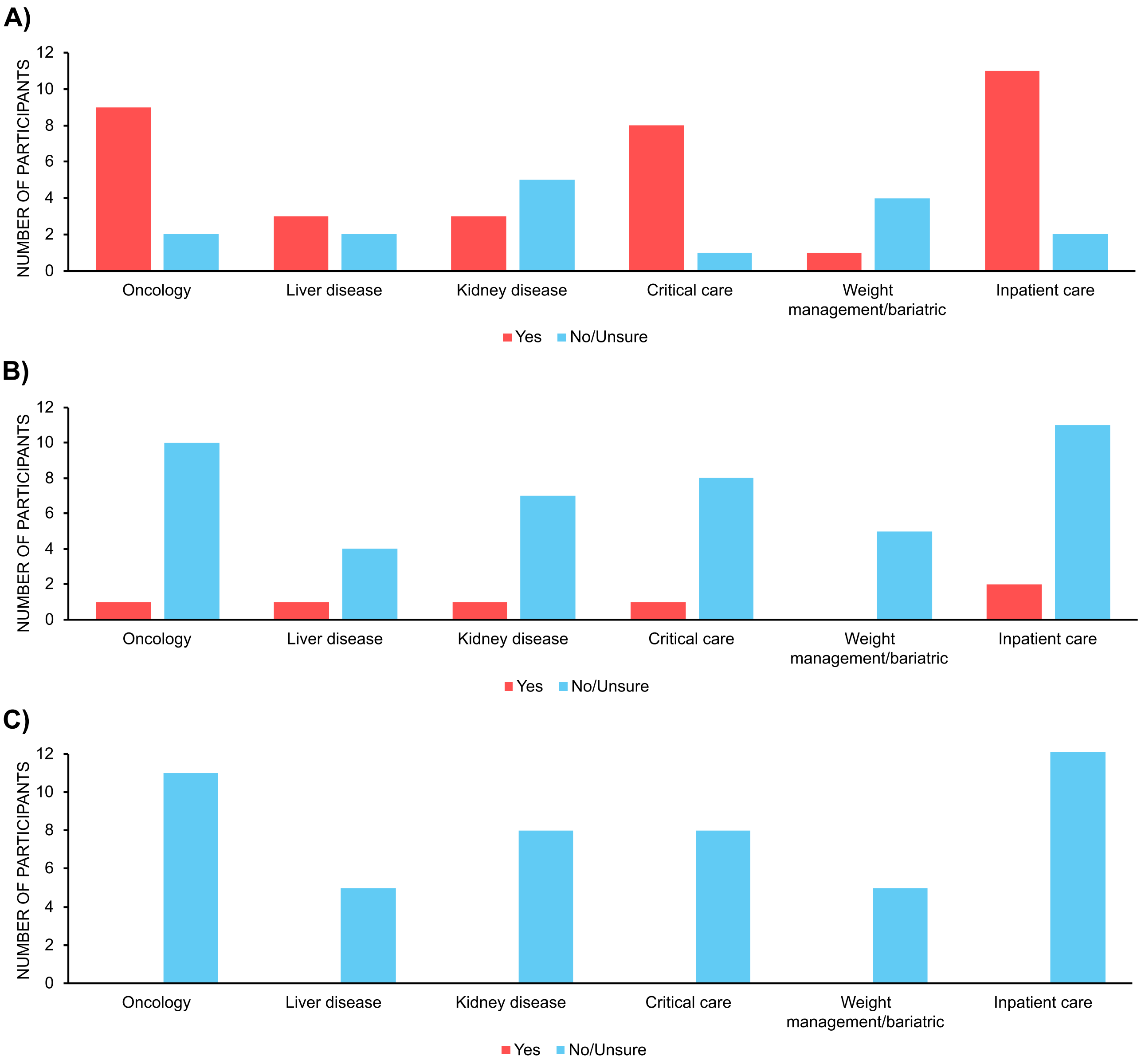

Figure S2. A) Participants' awareness of malnutrition screening and/or diagnostic protocols by practice area. **B)** Participants' awareness of sarcopenia screening and/or diagnostic protocols by practice area. **C)** Participants' awareness of sarcopenic obesity screening and/or diagnostic protocols by practice area. Note that because 41.7% of participants reported working in two or more practice areas, the total number of responses (n = 51) exceeds the total number of participants (N = 36).
